# Essential Nonredundant Function of the Catalytic Activity of Histone Deacetylase 2 in Mouse Development

**DOI:** 10.1128/MCB.00639-15

**Published:** 2016-01-19

**Authors:** Astrid Hagelkruys, Katharina Mattes, Verena Moos, Magdalena Rennmayr, Manuela Ringbauer, Anna Sawicka, Christian Seiser

**Affiliations:** Department of Medical Biochemistry, Max F. Perutz Laboratories, Medical University of Vienna, Vienna Biocenter, Vienna, Austria

## Abstract

The class I histone deacetylases (HDACs) HDAC1 and HDAC2 play partially redundant roles in the regulation of gene expression and mouse development. As part of multisubunit corepressor complexes, these two deacetylases exhibit both enzymatic and nonenzymatic functions. To examine the impact of the catalytic activities of HDAC1 and HDAC2, we generated knock-in mice expressing catalytically inactive isoforms, which are still incorporated into the HDAC1/HDAC2 corepressor complexes. Surprisingly, heterozygous mice expressing catalytically inactive HDAC2 die within a few hours after birth, while heterozygous HDAC1 mutant mice are indistinguishable from wild-type littermates. Heterozygous HDAC2 mutant mice show an unaltered composition but reduced associated deacetylase activity of corepressor complexes and exhibit a more severe phenotype than HDAC2-null mice. They display changes in brain architecture accompanied by premature expression of the key regulator protein kinase C delta. Our study reveals a dominant negative effect of catalytically inactive HDAC2 on specific corepressor complexes resulting in histone hyperacetylation, transcriptional derepression, and, ultimately, perinatal lethality.

## INTRODUCTION

Throughout development, reversible epigenetic mechanisms, including histone modifications, modulate gene expression in a tightly controlled manner to ensure proper differentiation and correct cell fate decisions. Histone deacetylases (HDACs) induce changes in the chromatin structure and thereby modulate the gene expression program. HDACs are crucial regulators of proliferation and differentiation, and inhibitors of these enzymes were shown to be promising drugs against malignant tumors and neurological diseases. Many HDAC inhibitors (HDACis) are under clinical investigation, and some of them are already approved for the treatment of lymphoma and bipolar disease due to their respective anticancer and mood-stabilizing effects (1). However, the exact mechanism of action of HDAC inhibitors is controversial and not yet clear. The Rpd3-like class I histone deacetylase members HDAC1 and HDAC2 display high amino acid sequence identity and are able to homo- and heterodimerize (reviewed in reference 2). HDAC1 and HDAC2 constitute the catalytic components of multisubunit repressor complexes, including the Sin3, NuRD, and CoREST complexes (3–7). Previous studies have shown that the two enzymes also exhibit noncatalytic functions in addition to their deacetylase activities (8–10).

To reveal the contribution of catalytic activity to the individual regulatory function of HDAC1/HDAC2 during mouse development, we generated knock-in mouse lines expressing catalytically inactive HDAC1/2 isoforms. While there is increasing knowledge of conventional HDAC1/2 knockout phenotypes and the underlying mechanisms, no study so far has explored the role of the enzymatic activity of HDAC1/HDAC2 in comparison to the structural function. Solving this question is particularly interesting for diseases, where HDACi treatment has been proven to be beneficial. In both cancer and disorders of the central nervous system, HDACs are promising targets for therapeutic intervention to reverse aberrant epigenetic changes and restore transcriptional balance (reviewed in references 11–14). Despite the beneficial therapeutic effects of HDACis in tumorigenesis and neurological diseases, the contribution of individual HDACs and the underlying molecular mechanisms of HDACi function are far from being fully understood. Since only very rare isoform-specific inhibitors exist, it would be important to mimic the situation of an isoform-specific HDACi treatment to more closely analyze the specific functions of different HDACs in health and disease. While conventional genetic deletion of HDACs results in their complete absence, eliminating both catalytic activity and their stabilizing function within corepressor complexes, we aimed to abolish only the enzymatic function, leaving the scaffolding function intact. In this way, the assembly and integrity of HDAC-containing multisubunit complexes are preserved, and the role of the enzymatic function of HDAC1/HDAC2 in comparison to a structural function can be explored. Here, we report that heterozygous expression of catalytically inactive HDAC2 has a dominant negative effect on the remaining wild-type (WT) HDAC2 enzyme and thereby leads to histone hyperacetylation and transcriptional derepression in the mouse brain, which eventually results in perinatal lethality. Strikingly, the phenotype of heterozygous inactive HDAC2 mutant mice is more severe than that of HDAC2-null mice. The heterozygous expression of catalytically inactive HDAC2 phenocopies a nervous system-specific deletion of 3 of the 4 *Hdac1/Hdac2* alleles, resulting in an upregulation of protein kinase C delta (PKCδ). Previous studies have shown regulatory functions for other class I deacetylases that do not depend on their catalytic activity (15, 16). This is, to our knowledge, the first report demonstrating that the catalytic activity of HDAC2 is absolutely required during mouse development.

## MATERIALS AND METHODS

### Animal care and transgenic mouse lines.

All experiments were performed in accordance with Austrian guidelines for animal care and protection authorized by the Austrian Ministry of Science and Research (protocol number GZ BMWFW-66.009/0113-WF/II/3b/2014). Chimeras were mated to C57BL/6 mice. *Hdac1*^KI/+^ mice were backcrossed for 3 to 5 generations on the C57BL/6 background (which did not affect the phenotype). Due to perinatal lethality, *Hdac2*^KI/+^ mice were not able to be backcrossed.

Mice were genotyped with the following primers: *Hdac1* knock-in genotyping primers AAGCAGCAGACGGACATC, TGAAGGAAGGTGGAAGAGTG, and GCATCGCATTGTCTGAGT and *Hdac2* knock-in genotyping primers GCTGGGGCTGTGAAATTAAACC, TCTGTGTAGAGGATGGATGAGAG, and ATGCGGTGGGCTCTATGG.

### Site-directed mutagenesis.

To introduce the His141→Ala point mutation into the *Hdac1* gene and the His142→Ala transition into the *Hdac2* gene, the GeneTailor site-directed mutagenesis system (Invitrogen) was used according to the manufacturer's instructions.

### Southern blot analysis.

Southern blot analysis was performed as previously described (17). Digested genomic DNA fragments were run on an agarose gel, depurinated in 0.25 M HCl, denatured (0.5 M NaOH, 1.5 M NaCl), and neutralized (0.5 M Tris-HCl [pH 7.4], 0.5 M NaCl) for 15 min each. The gel, membrane (GeneScreen; PerkinElmer), Whatman 1MM filter papers (Whatman), and a sponge were equilibrated in 10× SSC transfer buffer (150 mM trisodium citrate dihydrate, 1.5 M NaCl), and blotting was performed overnight. After UV cross-linking, successful blotting was checked by methylene blue staining, and blots were prehybridized in blocking solution (5× SSPE [1× SSPE is 0.18 M NaCl, 10 mM NaH_2_PO_4_, and 1 mM EDTA {pH 7.7}], 0.1 M sodium phosphate, 50% formamide, 5× Denhardt solution, 10 mM EDTA, 1% *N*-laurylsarcosine [sodium salt]) containing 200 μg/ml tRNA at 42°C for 2 h. The Stratagene Prime-It II Random Primer Labeling kit (Agilent Technologies) was used for radioactive labeling of 25 ng purified DNA probe with [α-^32^P]dCTP (10 mCi/ml; Hartmann Analytic) according to the manufacturer's protocol. The probe was subsequently cleaned up by using a Roche Quick Spin column according to the manufacturer's instructions (Roche), and specific activity was measured with a liquid scintillation analyzer (Packard). The purified labeled DNA probe was denatured at 95°C for 5 min, mixed with blocking solution, and put onto the hybridization sandwich. After overnight hybridization at 42°C, the blot was washed in 6× SSC–0.1% SDS and in 2× SSC–0.1% SDS at room temperature and finally washed in 0.2× SSC with 0.1% SDS at 55°C. After washing, the blot was wrapped in plastic foil, exposed to a PhosphorImager developer cassette, and scanned by using a Typhoon 8600 PhosphorImager (Molecular Dynamics/Amersham Biosciences) after exposure.

### RNA isolation and quantitative reverse transcription-PCR (qRT-PCR) analysis.

Brains were isolated and homogenized in TRIzol reagent (Invitrogen). Total RNA was isolated according to the manufacturer's instructions. RNA was reverse transcribed with the iScript cDNA synthesis kit (Bio-Rad). Real-time PCR analysis was performed with Kapa SYBR Fast qPCR master mix (Peqlab) on an iCycler IQ system (Bio-Rad). Data were normalized to values for the housekeeping gene *Gapdh* or *Hprt*.

The following primers were used: ATCTACCGTCCTCACAAAGC and CTTCAGACTTCTTTGCATG for WT *Hdac1*, ATCTACCGTCCTCACAAAGC and CTTCAGACTTCTTTGCGGC for the *Hdac1* mutant, GCCACTGCTGAAGAAATG and GCTTCTGACTTCTTGGCATG for WT *Hdac2*, GCCACTGCTGAAGAAATG and GCTTCTGACTTCTTGGCGGC for the *Hdac2* mutant, GCTGGTGAAAAGGACCTCT and CACAGGACTAGAACACCTGC for *Hprt*, GTCGGTGTGAACGGATTTGG and GACTCCACGACATACTCAGC for *Gapdh*, TGATGACAAGGAGGAGTATG and CGGTGACGCAGAAGAG for *Myh6*, AGACGGAGAATGGCAAGAC and GACGGTGACGCAGAAGAG for *Myh7*, GGCTATTCCTTCGTGAC and CCGCAGACTCCATACC for *Acta1*, GCCGTGTTATCCAGATTGTG and TCAGCCTTGCCGTTGTTC for *Prkcd*, ACACATTCTGGCTCACG and GCGGTTATTGCGAAGG for *Dmc1*, GAGCAGATGAGTGATG and TGACAGCCGAAGAAG for *Echdc2*, TGTTCCTGTTCTTCCTCGATG and TCTAACTGCCTGGTCTGTG for *Edn1*, TGACCACAAGCACCACTG and CGGCACTTCATCCTCCTG for *Ppap2c*, CCAGTGACCAAGATGTAG and AATACCGCAAACCAAGG for *Rom1*, and ACTGCTGCTGCCACTACTG and ACTCCGATTCCTCTTATTGATTGC for *Tcfl5*.

### RNA-seq and data analysis.

For transcriptome sequencing (RNA-seq) experiments, RNA was subjected to poly(A) selection with a Dynabeads mRNA purification kit (Invitrogen), followed by reverse transcription using a NEB RNA Ultra kit and library generation using a TruSeq library generation kit (Illumina). We performed full-length mRNA-seq experiments in three biological replicates for *Hdac2*^KI/+^ and the corresponding wild-type brains and applied the “union” model of the htseq-count script (18) to calculate the number of reads associated with each of the 21,608 mouse RefSeq genes for each sample. We used these counts to compute reads per kilobase per million (RPKM) values for each gene and determined Spearman's correlation coefficient (ρ) for each set of biological replicates. Based on the high correlation of the replicates (ρ = 0.99 between each 2 of the 3 wild-type brains and between each 2 of the 3 *Hdac2*^KI/+^ brains), we used the log-transformed means of RPKM values under each condition to plot the distribution of gene expression levels by using kernel density estimation. Based on this distribution, we set the threshold for gene expression to 1 RPKM (log_2_ RPKM value equal to zero). This is consistent with data from previous studies, which estimated that the value of 1 RPKM corresponds to 1 transcript per cell (19). The analysis of differentially expressed genes across the two conditions was performed by using htseq-count and the Bioconductor edgeR package (20, 21). Genes that showed a minimum of a 2-fold change in expression levels (adjusted *P* value of ≤0.05) were classified as upregulated, whereas genes displaying a fold change of ≤0.5 (adjusted *P* value of ≤0.05) were categorized as downregulated.

### Chromatin immunoprecipitation and PCR analysis.

Isolated brains were finely chopped, washed with phosphate-buffered saline (PBS), and cross-linked with disuccinimidyl glutarate (DSG) (2 mM; AppliChem) for 25 min at room temperature. After another PBS washing step, the brains were cross-linked by the addition of formaldehyde (to a final concentration of 1%) at room temperature for 10 min. The cross-linking process was stopped by the addition of 125 mM glycine. The chromatin isolation procedure was performed as previously described (22). For chromatin immunoprecipitation (ChIP), equal amounts of sonicated chromatin were diluted 10-fold and precipitated overnight with the following antibodies: HDAC1 (Sat13; Seiser Laboratory), HDAC2 (Bethyl Laboratories), H3K9ac (Millipore), H4ac (Millipore), C-terminal H3 (clone 1B1-B2; Active Motif), and rabbit IgG (Invitrogen) as a control. Chromatin-antibody complexes were isolated by using protein A beads for rabbit primary antibodies or protein G beads for mouse primary antibodies (Dynabeads; Invitrogen). PCRs with 1:20 dilutions of genomic DNA (input) and with the precipitated DNA were carried out. The extracted DNA was used for quantitative PCR analysis with the primers listed below. ChIP signals for histone modifications were normalized to the H3 C-terminal signal to correct for changes in nucleosomal density.

The following primers were used: Prkcd(−2322) (TCTAGAGCTCCCCAATGGCT and CACCCTATCTGGCTCCTCCT), Prkcd(−771) (CCCAGCCCAGGAAGTCATTT and CCCTTGCTCAGTCAAGCTCA), Prkcd(−251) (GTCCTCCTCTATAAATTAGTCC and TCAGCCTCTTTGAGTTGC), Prkcd(304) (CTCGCCCGTAGTCTCCATTC and GGCGGGGATAAAGACACACA), Prkcd(4999) (CCTGCCCTTGTGCAAAACTC and TCATAGCAGGGAGCCTCTGT), and Prkcd(24069) (GGTGTTGATTGACGATGATG and AAGCAGAGGTAAAGGGTAAG).

### Protein isolation, immunoblot analysis, and HDAC activity assays.

Dissected brains were immediately frozen in liquid nitrogen and stored at −80°C. For protein extraction, frozen brains were manually homogenized in Hunt buffer (20 mM Tris-HCl [pH 8.0], 100 mM sodium chloride, 1 mM EDTA, 0.5% NP-40) supplemented with protease inhibitor cocktail (Roche) and phenylmethylsulfonyl fluoride (PMSF) in a glass homogenizer. After full-speed centrifugation, the supernatant containing the soluble protein fraction was further used. Equal amounts of 20 to 30 μg of proteins were separated by SDS-PAGE (10% gels) and transferred onto nitrocellulose membranes (Protran; Whatman) according to standard protocols. For detection, the Enhanced Chemiluminescence kit (PerkinElmer) was used. HDAC activity assays were performed with brain protein extracts as previously described (9). Primary antibodies for immunoblotting were HDAC1 (10E2 or Sat13), HDAC2 (3F3), Sin3A (catalog number sc-994; Santa Cruz), CoREST (catalog number 07-455; Millipore), MTA1 (catalog number sc-9446; Santa Cruz), PKCδ (catalog number 610397; BD), and β-actin (catalog number A5316; Sigma) antibodies.

### Coimmunoprecipitation assay.

Total protein extracts from brain were harvested as described above. Equal amounts of 1 mg of protein were incubated for 1 h at 4°C with 4 μg antibody. Immunoprecipitation was carried out by using protein A beads or protein G beads (Dynabeads; Invitrogen) overnight at 4°C. The immune complexes were washed three times with Hunt buffer. Samples were used for an HDAC activity assay, or they were heated in SDS sample buffer and used for immunoblotting. Primary antibodies used for coimmunoprecipitation were Sin3A (catalog number sc-994X; Santa Cruz), CoREST (catalog number 07-455; Millipore), and MTA1 (catalog number sc-9446; Santa Cruz) antibodies.

### Histone immunoblot analysis.

Dissected brains were immediately frozen in liquid nitrogen and stored at −80°C. Frozen brains were manually homogenized in lysis buffer (10 mM Tris-HCl [pH 6.5], 50 mM sodium disulfite, 10 mM MgCl_2_, 10 mM sodium butyrate, 8.6% sucrose, 1% Triton X-100) supplemented with protease inhibitor cocktail (Roche) and PMSF. Histone isolation was performed as previously described (23). Equal amounts of histones (2 μg) were separated by SDS-PAGE and transferred onto nitrocellulose membranes (Protran; Whatman) according to standard protocols. The following antibodies were used: H3 C-terminal (catalog number ab1791; Abcam), H3K14ac (catalog number 07-353; Millipore), H3K27ac (catalog number ab4729; Abcam), H3K4ac (catalog number 39381; Active Motif), H3K56ac (catalog number ab76307; Abcam), H3K9ac (catalog number 06-942; Millipore), H4K12ac (Sat44; Seiser laboratory), H4K16ac (Sat53; Seiser laboratory), and H4K8ac (Sat198; Seiser laboratory) antibodies.

### Histological and IHC analyses.

Tissue samples were fixed overnight in 4% paraformaldehyde and further embedded in paraffin. All stainings were performed on 4-μm sections. Stainings with hematoxylin and eosin (H&E) were carried out according to standard procedures with an ASS1 staining unit (Pathisto). Fluorescence stainings were performed with the DyLight system (ThermoScientific) or the Tyramide Signal Amplification kit (PerkinElmer), according to the manufacturer's instructions. The slides were counterstained with 4′,6-diamidino-2-phenylindole (DAPI) and mounted in ProLong Gold (Invitrogen).

The primary antibody used for immunohistochemistry (IHC) was H3S10ph antibody (catalog number sc-8656; Santa Cruz).

### Microscopy.

H&E-stained samples were imaged on a Zeiss stereomicroscope with a camera. Images of IHC fluorescence stainings were captured on an LSM Meta 710 confocal microscope (Zeiss). The cerebellum perimeter and the cortex area of H&E-stained brain sections were quantified by using ImageJ.

### Statistical analysis.

Real-time PCR and chromatin immunoprecipitation experiments were evaluated with Microsoft Excel. The relative intensities of bands detected in immunoblots were estimated by using ImageQuant software, and relative protein expression levels were normalized to β-actin values. The significance between groups was determined by the unpaired (two-tailed) Student *t* test. *P* values were calculated with GraphPad Prism software, and standard deviations (SD) are shown.

## RESULTS

### Perinatal lethality of mice expressing inactive HDAC2.

**FIG 1:**
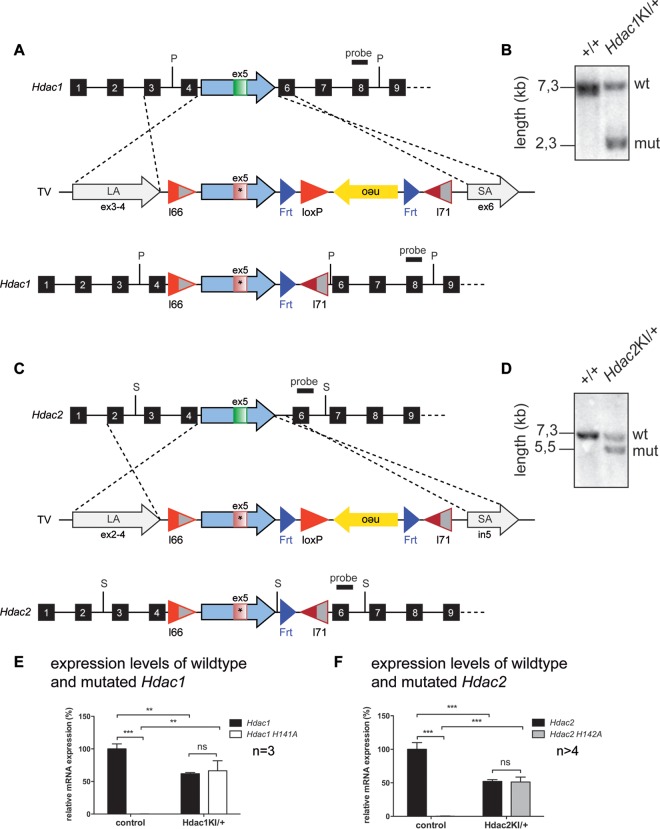
Successful ES cell targeting of the *Hdac1/Hdac2* allele leads to expression of catalytically inactive HDAC1/2 in the heterozygous state. (A and C) The targeting vectors (TV) contain a long arm (LA) and a short arm (SA) of homology, allowing homologous recombination in ES cells and resulting in the insertion of the targeting cassette into the *Hdac1/Hdac2* gene. The introduced cassette contains the mutated version of *Hdac1/Hdac2* exon 5 (red exon with an asterisk) and substitutes endogenous wild-type exon 5 (green). After FlpE recombinase-mediated deletion, the *neo* cassette and an additional *loxP* site are removed. Exons are depicted as black boxes, and the locations of the restriction enzymes (P, PstI; S, SacI) and probes for Southern blot analyses are shown. (B and D) Southern blot analysis of control wild-type and heterozygous *Hdac1*^KI/+^ (B) and *Hdac2*^KI/+^ (D) ES cells. mut, mutant. (E) Relative mRNA expression levels of wild-type *Hdac1* and mutated *Hdac1*-H141A in *Hdac1*^KI/+^ brains compared to the corresponding wild-type littermate controls. Values are normalized to the values for the housekeeping gene *Hprt*. Error bars indicate SD (*n* = 3). **, *P* < 0.01; ***, *P* < 0.001; ns, not significant. (F) Relative mRNA expression levels of wild-type *Hdac2* and mutated *Hdac2*-H142A in *Hdac2*^KI/+^ brains compared to the corresponding wild-type littermate controls. Values are normalized to the values for the housekeeping gene *Hprt*. Error bars indicate SD (*n* > 4). ***, *P* < 0.001.

**FIG 2:**
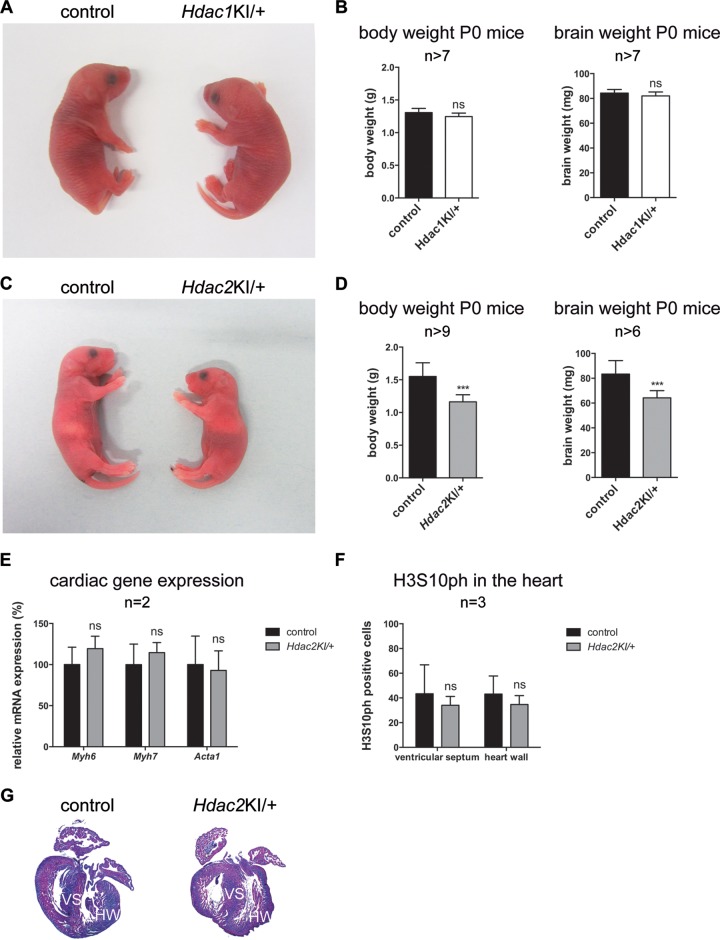
Divergent phenotypes upon heterozygous expression of HDAC1-H141A and HDAC2-H142A. (A) Representative picture of a wild-type newborn mouse (left) versus an *Hdac1*^KI/+^ newborn littermate (right). (B) Body and brain weights of P0 control (black) compared to *Hdac1*^KI/+^ (white) mice. Error bars indicate SD (*n* > 7). ns, not significant. (C) Representative pictures of a wild-type newborn mouse (left) versus an *Hdac2*^KI/+^ newborn littermate (right). (D) Body and brain weights of P0 control (black) compared to *Hdac2*^KI/+^ (gray) mice. Error bars indicate SD (*n* > 9 [left] and *n* > 6 [right]). ***, *P* < 0.001. (E) Relative mRNA expression levels of *Myh6*, *Myh7*, and *Acta1* in *Hdac2*^KI/+^ hearts compared to the corresponding wild-type littermate controls. Values are normalized to the values for the housekeeping gene *Gapdh*. Error bars indicate SD (*n* = 2). ns, not significant. (F) H3S10ph-positive cells in the ventricular septum and heart wall in *Hdac2*^KI/+^ P0 mice compared to the corresponding wild-type littermate controls. Error bars indicate SD (*n* = 3). ns, not significant. (G) H&E-stained P0 wild-type control littermate (left) and *Hdac2*^KI/+^ (right) paraffin heart sections. HW, heart wall; VS, ventricular septum.

To assess the enzymatic function of HDAC1 and HDAC2 *in vivo*, we generated mice expressing enzymes with a single-amino-acid substitution from histidine to alanine at position 141 (HDAC1-H141A) and HDAC2-H142A, respectively. This mutation in the catalytic center of the enzyme was shown to strongly reduce the enzymatic activity while not affecting the interaction with components of the HDAC1/HDAC2 corepressor complexes (24). Therefore, targeting vectors were constructed and inserted into the respective wild-type *Hdac1* and *Hdac2* genes of mouse embryonic stem (ES) cells by homologous recombination (Fig. 1A to D). Successfully targeted A9 ES cells were used for blastocyst injection. All of the injected clones gave rise to chimeric mice, which showed germ line transmission, and for each of the constructs, two chimeric mice derived from independent ES cell clones were chosen for breeding with C57BL/6 wild-type mice. Heterozygous knock-in mice expressing HDAC1-H141A and HDAC2-H142A (here referred to as *Hdac1*^KI/+^ and *Hdac2*^KI/+^ mice, respectively) were born at the expected ratios. Heterozygous *Hdac1*^KI/+^ mice were viable and fertile and displayed no obvious phenotype (Fig. 2A and B), while from several *Hdac1*^KI/+^ × *Hdac1*^KI/+^ crossings, 52% wild-type and 48% heterozygous *Hdac1*^KI/+^ but no *Hdac1*^KI/KI^ pups were born (total, *n* = 25). These data indicate that heterozygous expression of catalytically inactive HDAC1 has no effect on mouse embryogenesis, whereas homozygous HDAC1-H141A expression might cause embryonic lethality similarly to the complete ablation of HDAC1 (25–27).

In contrast, all heterozygous *Hdac2*^KI/+^ mice died within several hours after birth with complete penetrance (*n* > 45) and showed absent milk bellies and reduced body and brain weights (Fig. 2C and D). This is also opposite from heterozygous HDAC2 knockout (*Hdac2*^+/−^) mice, which have been reported to show normal development (28). Heterozygous *Hdac1*^KI/+^ or *Hdac2*^KI/+^ mice showed the expected expression of 50% wild-type and 50% mutant *Hdac1/Hdac2* mRNA levels (Fig. 1E and F). Given that HDAC1 is the more important enzyme during embryogenesis (29), it is surprising that heterozygous HDAC1-H141A mice display no obvious phenotype, while heterozygous HDAC2-H142A animals show perinatal lethality. Therefore, we aimed to characterize heterozygous HDAC2-H142A-expressing mice in more detail.

Different phenotypes and a spectrum of cardiac defects have been reported for conventional deletion of *Hdac2*. One study reported that mice lacking HDAC2 exhibit complete lethality within 24 h after birth and severe heart defects, including increased cardiac proliferation and apoptosis (26). However, several other studies showed that HDAC2-deficient mice are at least in part viable (28, 30–32). One study demonstrated the lethality of 50% of HDAC2 knockout mice during the first 25 postnatal days due to alterations of fetal cardiac isoform gene expression and a thickened myocardium (31). Interestingly, the surviving mice recovered and did not show differences from wild-type littermates in adult age. The different outcomes of these studies might be due to different knockout strategies and genetic backgrounds (discussed in references 26 and 30).

Therefore, we checked heart anatomy, cardiac cell proliferation, and fetal cardiac isoform gene expression levels, which were shown to be altered upon the loss of HDAC2. In contrast to HDAC2-deficient mice (26, 31), heterozygous HDAC2-H142A-expressing mice did not exhibit heart defects or changes in cardiac cell proliferation and fetal cardiac isoform gene expression (Fig. 2E to G), indicating that the lethality is not caused by cardiac defects.

### Dominant negative effect of inactive HDAC2.

**FIG 3:**
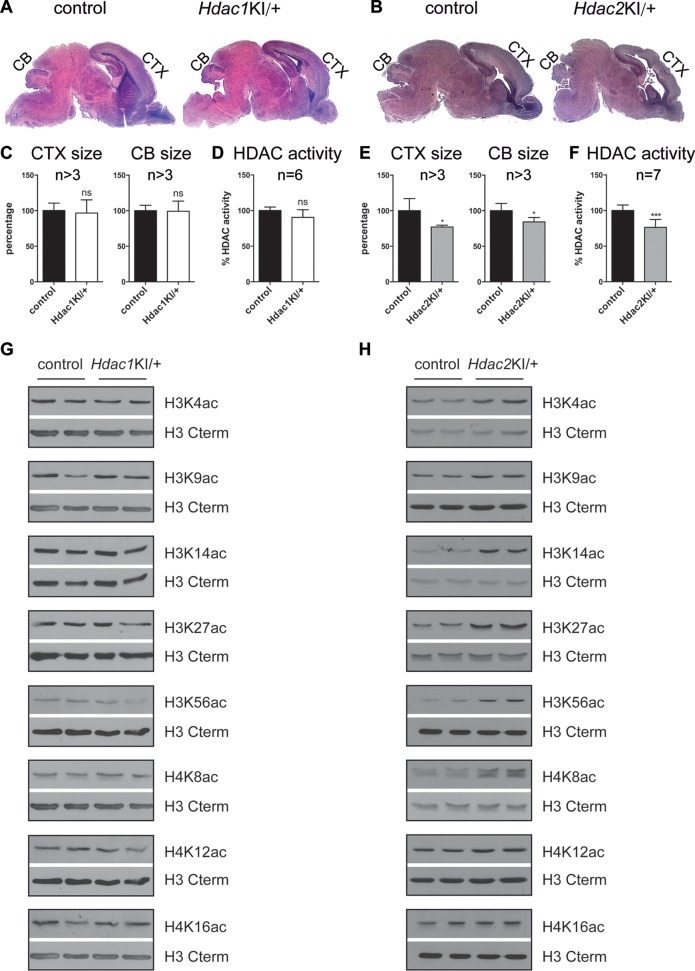
Changes in brain architecture, cellular deacetylase activity, and histone acetylation patterns upon expression of catalytically inactive HDAC2. (A and B) H&E-stained paraffin sections from P0 wild-type control littermates versus *Hdac1*^KI/+^ (A) and *Hdac2*^KI/+^ (B) mice. CB, cerebellum; CTX, cortex. (C and E) Quantification of P0 cortex and cerebellum sizes in *Hdac1*^KI/+^ (C) and *Hdac2*^KI/+^ (E) brains. Error bars indicate SD (*n* > 3). *, *P* < 0.05; ns, not significant. (D and F) Total histone deacetylase activity in P0 *Hdac1*^KI/+^ (D) and *Hdac2*^KI/+^ (F) brains. Error bars indicate SD (*n* = 6 [D] and *n* = 7 [F]). ***, *P* < 0.001; ns, not significant. (G and H) Representative immunoblot analyses of histones prepared from two P0 *Hdac1*^KI/+^ brains (G) and two P0 *Hdac2*^KI/+^ brains (H) versus two wild-type littermate controls. The membranes were probed with antibodies against H3K4ac, H3K9ac, H3K14ac, H3K27ac, H3K56ac, H4K8ac, H4K12ac, and H4K16ac, and for each blot, the H3 C-terminal antibody was used as a loading control.

The brains of heterozygous *Hdac1*^KI/+^ mice are indistinguishable in size and architecture from those of wild-type littermates (Fig. 3A and C). In contrast, brains of heterozygous *Hdac2*^KI/+^ mice were smaller and more fragile and showed reduced sizes of the cortex and cerebellum and a diminished foliation of the cerebellum (Fig. 3B and E). Interestingly, time of death and brain architecture of heterozygous HDAC2-H142A-expressing mice were reminiscent of those of mice with a nervous system-specific deletion of both *Hdac2* alleles and one additional *Hdac1* allele (*Hdac1*^Δ/+n^ *Hdac2*^Δ/Δn^), as described previously (9). This prompted us to analyze the brains of *Hdac2*^KI/+^ mice in more detail.

Given the surprising phenotype of *Hdac2*^KI/+^ mice, we analyzed the impact of HDAC2-H142A expression on total cellular HDAC activity in mutant brains. Total cellular HDAC activity was reduced to 75% in *Hdac2*^KI/+^ brains (Fig. 3F) but was not significantly changed in *Hdac1*^KI/+^ brains (Fig. 3D). Accordingly, the abundances of several histone acetylation marks, including H3K4ac, H3K14ac, H3K27ac, H3K56ac, and H4K8ac, were increased in *Hdac2*^KI/+^ brains but not in *Hdac1*^KI/+^ brains (Fig. 3G and H).

**FIG 4:**
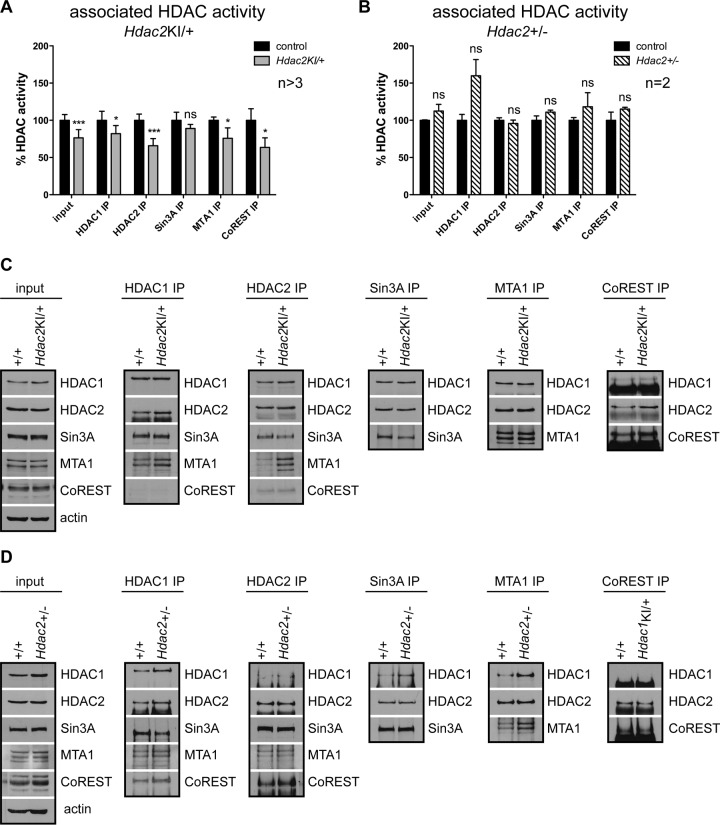
Dominant negative effect of catalytically inactive HDAC2-H142A. Shown are data from coimmunoprecipitation experiments with P0 brain protein extracts from *Hdac2*^KI/+^ (A and C) and *Hdac2*^+/−^ (B and D) mice and the corresponding wild-type littermate controls using antibodies against HDAC1, HDAC2, Sin3A, MTA1, and CoREST. (A and B) Total deacetylase activity was analyzed in inputs and immunoprecipitates (IP). Error bars indicate SD (*n* > 3 [A] and *n* = 2 [B]). *, *P* < 0.05; ***, *P* < 0.001; ns, not significant. (C and D) The same input extracts and immunoprecipitates from *Hdac2*^KI/+^ (C) and *Hdac2*^+/−^ (D) mice with the corresponding wild-type littermate controls were analyzed in immunoblots with antibodies against HDAC1, HDAC2, CoREST, Sin3A, MTA1, and β-actin, which was used as a loading control.

To compare the effects of partial HDAC2 inactivation with those of reduced HDAC2 expression, we examined the deacetylase activities associated with HDAC1, HDAC2, and HDAC1/HDAC2 corepressor complexes and performed coimmunoprecipitation experiments for HDAC1, HDAC2, CoREST, Sin3A, and MTA1 (NuRD) with brain extracts from *Hdac2*^KI/+^ mice, *Hdac2*^+/−^ mice, and the corresponding wild-type littermates (Fig. 4). As expected, expression of HDAC2-H142A in the presence of wild-type HDAC2 led to reduced HDAC2-associated deacetylase activity (66% of the wild-type control) (Fig. 4A). HDAC1 and HDAC2 are able to homo- and heterodimerize (24, 33). Thus, part of the HDAC2-associated deacetylase activity is contributed by coprecipitated HDAC1 and vice versa. This might explain why HDAC2-associated deacetylase activity is not reduced to 50% despite the equal expression of HDAC2 wild-type and mutant proteins (data not shown). Accordingly, HDAC1-associated deacetylase activity is also slightly but significantly reduced in the presence of inactive HDAC2 protein in *Hdac2*^KI/+^ brains compared to wild-type brains (Fig. 4A). In contrast, the loss of one *Hdac2* allele (*Hdac2*^+/−^) did not reduce the deacetylase activities associated with HDAC1, HDAC2, or HDAC1/2-containing corepressor complexes in the brain (Fig. 4B).

As shown in Fig. 4C, the expression of the inactive HDAC2 isoform did not affect incorporation into the Sin3A, NuRD (MTA1), and CoREST multisubunit corepressor complexes. However, we observed a significant reduction of CoREST- and NuRD-associated HDAC activity upon heterozygous expression of inactive HDAC2 (Fig. 4A), while the loss of a single *Hdac2* allele (*Hdac2*^+/−^) had no effect on corepressor-associated deacetylase activity (Fig. 4B). Taken together, the expression of catalytically inactive HDAC2-H142A has a dominant negative effect on the deacetylase activity of specific HDAC1/HDAC2 corepressor complexes.

### Transcriptional derepression by enzymatically inactive HDAC2.

**FIG 5:**
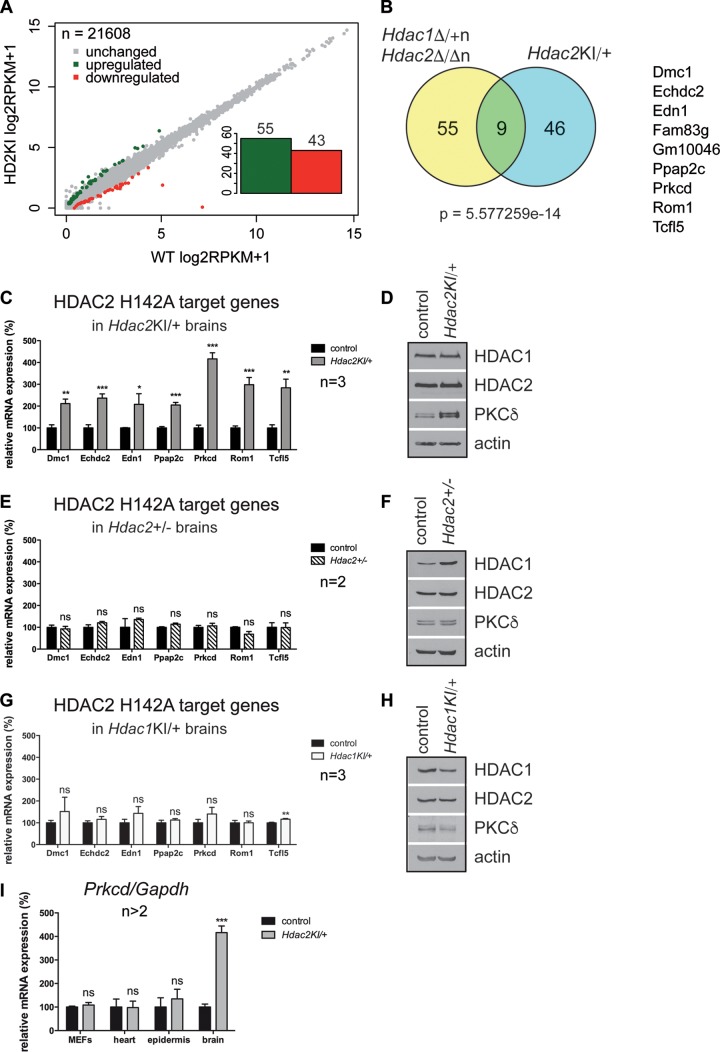
Transcriptional derepression and upregulation of protein kinase C delta in *Hdac2*^KI/+^ brains. (A) Changes in mRNA abundances of 21,608 RefSeq genes upon heterozygous expression of HDAC2-H142A in P0 *Hdac2*^KI/+^ brains determined by full-length mRNA-seq (3 *Hdac2*^KI/+^ brains versus 3 wild-type control littermate brains at P0). (B) Venn diagram of upregulated genes (fold change of ≥2; *P* value of <0.05) in *Hdac1*^Δ/+n^ *Hdac2*^Δ/Δn^ brains (yellow) (9) compared to *Hdac2*^KI/+^ brains (blue). The overlap (green) includes the 9 genes shown on the right. The *P* value (5.6 × 10^−14^) was calculated by using a hypergeometric test. (C, E, and G) Relative mRNA expression levels of *Dmc1*, *Echdc2*, *Edn1*, *Ppap2c*, *Prkcd*, *Rom1*, and *Tcfl5* in P0 *Hdac2*^KI/+^ brains (C), *Hdac2*^+/−^ brains (E), and *Hdac1*^KI/+^ brains (G) compared to the corresponding wild-type littermate controls. Values are normalized to the values for the housekeeping gene *Gapdh*. Error bars indicate SD (*n* = 3 [C and G] and *n* = 2 [E]). *, *P* < 0.05; **, *P* < 0.01; ***, *P* < 0.001; ns, not significant. (D, F, and H) Immunoblot analyses of P0 wild-type littermate control versus *Hdac2*^KI/+^ brain extracts (D), *Hdac2*^+/−^ brain extracts (F), and *Hdac1*^KI/+^ brain extracts (G), with antibodies against HDAC1, HDAC2, PKCδ, and β-actin, which was used as a loading control. (I) Relative mRNA expression levels of *Prkcd* in different *Hdac2*^KI/+^ tissues compared to the corresponding wild-type littermate controls. Values are normalized to the values for the housekeeping gene *Gapdh*. Error bars indicate SD (*n* > 2). ns, not significant; ***, *P* < 0.001. MEFs, mouse embryonic fibroblasts.

To gain insight into changes in transcript abundance upon the expression of enzymatically inactive HDAC2 in the heterozygous state, we performed a differential expression analysis of full-length mRNA-seq data for brains of postnatal day 0 (P0) *Hdac2*^KI/+^ mice and the corresponding wild-type littermates. We identified 98 differentially expressed genes, 55 of which showed an increase in their mRNA levels in *Hdac2*^KI/+^ brains (fold change of ≥2; *P* value of <0.05) (Fig. 5A; see also the supplemental material). Given the similar phenotypes of *Hdac2*^KI/+^ mice and *Hdac1*^Δ/+n^ *Hdac2*^Δ/Δn^ mice, we compared the deregulated genes of *Hdac2*^KI/+^ and *Hdac1*^Δ/+n^ *Hdac2*^Δ/Δn^ brains. Remarkably, we detected a significant overlap (*P* value of 5.6 × 10^−14^, as determined by a hypergeometric test) between the 64 upregulated genes in *Hdac1*^Δ/+n^ *Hdac2*^Δ/Δn^ brains and the 55 upregulated genes in *Hdac2*^KI/+^ brains (fold change of ≥2; *P* value of <0.05) (Fig. 5B). The set of commonly upregulated genes included *Dmc1*, *Echdc2*, *Edn1*, *Fam83g*, *Gm10046*, *Ppap2c*, *Prkcd*, *Rom1*, and *Tcfl5*. Some of these genes have known functions in recombination (*Dmc1*), transcription control (*Tcfl5*), and signaling (*Edn1*, *Ppap2c*, and *Prkcd*). Overexpression of these potential target genes was confirmed by qRT-PCR (Fig. 5C). Importantly, deregulation of these genes in the mouse brain was caused specifically by the expression of the inactive HDAC2-H142A isoform, since neither the loss of a single *Hdac2* allele (*Hdac2*^+/−^) nor the expression of HDAC1-H141A (*Hdac1*^KI/+^) affected the expression of this set of genes (Fig. 5E and G). Together, the data indicate a specific dominant negative effect of the inactive HDAC2 protein and highlight the importance of HDAC2's enzymatic function during brain development.

Of the 9 commonly upregulated target genes in *Hdac2*^KI/+^ and *Hdac1*^Δ/+n^ *Hdac2*^Δ/Δn^ brains, *Prkcd* was 4-fold upregulated on the RNA level and 3.5-fold upregulated (*n* = 4; *P* = 0.0002) on the protein level (Fig. 5C and D). *Prkcd* encodes protein kinase C delta (PKCδ). This Ser/Thr kinase is a crucial regulator of proliferation, differentiation, apoptosis, autophagy, and energy metabolism in mammalian cells (34, 35). In *Hdac1*^Δ/+n^ *Hdac2*^Δ/Δn^ brains, the overexpression of PKCδ resulted in decreased proliferation and premature differentiation (9). In *Hdac2*^KI/+^ mice, PKCδ overexpression was limited to brains and was not observed in other *Hdac2*^KI/+^ tissues and cell types (Fig. 5I) or in *Hdac2*^+/−^ and *Hdac1*^KI/+^ mice (Fig. 5F and H). Interestingly, phosphatidic phosphatase type 2C (PPAP2C), which can convert phosphatidic acid to diacylglycerol and thereby activate PKCδ in a diacylglycerol-dependent manner, was also found at increased levels in *Hdac2*^KI/+^ brains.

**FIG 6:**
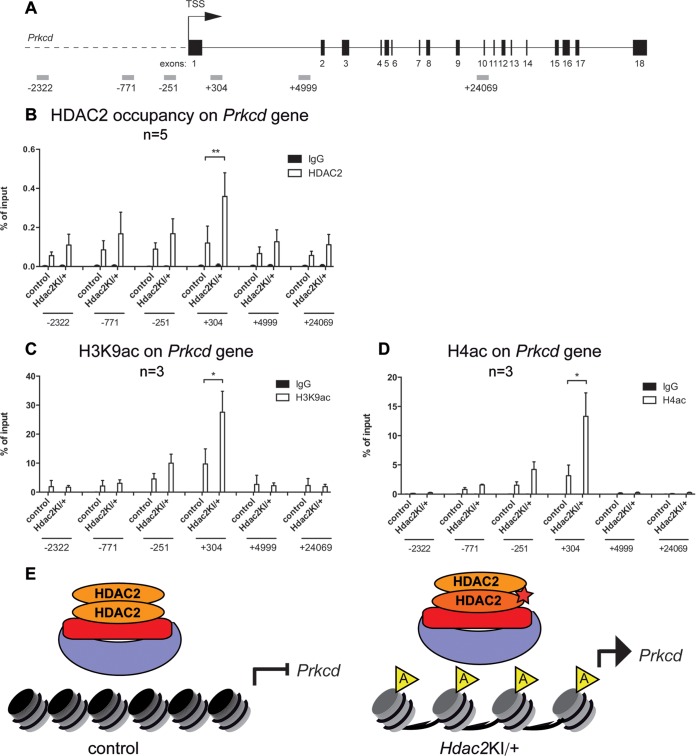
HDAC2 association and histone hyperacetylation at the *Prkcd* gene in *Hdac2*^KI/+^ brains. (A) Schematic representation of the *Prkcd* gene, with exons depicted as numbered black boxes. Primers used for the chromatin immunoprecipitation experiment are illustrated as gray rectangles. TSS, transcriptional start site. (B to D) Chromatin from littermate control and *Hdac2*^KI/+^ brains was immunoprecipitated with antibodies specific for HDAC2 (B), H3K9ac (C), H4ac (D), and IgG as a negative control, followed by qRT-PCR with primers specific for different regions of the *Prkcd* gene, as illustrated in panel A. Error bars indicate SD (*n* = 5 [B] and *n* = 3 [C and D]). For the region of interest (position +304), significance was tested with a *t* test. *, *P* < 0.05; **, *P* < 0.01. Note that in most cases, the IgG negative control has a very low percentage of input levels and is therefore not visible in most of the graphs. (E) Model of *Prkcd* overexpression. (Left) In the P0 wild-type brain, HDAC2 efficiently deacetylates nucleosomes at the *Prkcd* gene and thereby represses its transcription. (Right) In the *Hdac2*^KI/+^ brain, recruitment of catalytically inactive HDAC2 results in histone hyperacetylation and premature *Prkcd* expression.

To test if the *Prkcd* gene is a direct target of HDAC2 and to assess the histone acetylation levels of the *Prkcd* promoter in *Hdac2*^KI/+^ brains, we performed site-directed chromatin immunoprecipitation (ChIP) experiments with antibodies specific for HDAC2 and the histone marks H3K9ac and H4ac, which are known substrates of HDAC1/HDAC2 (36), in different regions of the *Prkcd* gene locus (Fig. 6A). In both control wild-type and *Hdac2*^KI/+^ brains, HDAC2 was associated with regions surrounding exon 1 of the *Prkcd* gene (Fig. 6B). This region contains conserved CG boxes that are crucial for the transcriptional upregulation of the *Prkcd* gene by HDAC inhibitors (37, 38).

Despite the enhanced presence of HDAC2, acetylation of H3K9 and H4 was markedly increased in *Hdac2*^KI/+^ brains, indicating the consequence of inactive HDAC2-H142A expression in the heterozygous state (Fig. 6C and D). Thus, the recruitment of inactive HDAC2-H142A results in histone hyperacetylation and premature *Prkcd* expression in *Hdac2*^KI/+^ brains, as summarized in the model shown in Fig. 6E.

## DISCUSSION

In this study, we have examined the impact of the catalytic activity of HDAC1 and HDAC2 on mouse development by using knock-in mice. In several previous reports, enzymatically inactive HDAC1 isoforms were used in overexpression experiments in the presence of their active wild-type counterpart (24, 33). However, to our knowledge, no study explored the role of the enzymatic function of HDAC1 and HDAC2 in comparison to their structural function *in vivo* in a mouse model. This is particularly interesting for neurological diseases and cancer, where HDAC inhibitor treatment has proven to be beneficial.

Our data demonstrate that a catalytically inactive HDAC2 isoform that is still incorporated into corepressor complexes has a dominant negative function on corepressor activity and mouse development. In contrast, heterozygous expression of HDAC1-H141A does not lead to a major phenotype. This is remarkable, since HDAC1 is essential for early mouse development, and HDAC1-null mice have a much more severe phenotype than that of HDAC2-null mice (26, 29–31). Importantly, heterozygous expression of catalytically inactive HDAC2 also has a more severe phenotype than the heterozygous ablation of *Hdac2* (no phenotype) or even the complete loss of HDAC2. As previously reported (28, 30–32), we observed that 50% of all HDAC2-null mice are viable and fertile (A. Hagelkruys and C. Seiser, unpublished observations). None of the cardiac defects that have been reported upon conventional deletion of *Hdac2* (26, 31) were found in HDAC2-H142A-expressing *Hdac2*^KI/+^ mice. However, we noticed altered brain architecture upon heterozygous expression of HDAC2-H142A (Fig. 3). Interestingly, the time of death, phenotype, and brain architecture are highly reminiscent of those of mice with a nervous system-specific deletion of two *Hdac2* alleles and one additional *Hdac1* allele (*Hdac1*^Δ/+n^ *Hdac2*^Δ/Δn^), as described previously (9). The H141A/H142A mutation has been shown to result in significantly decreased enzymatic activity without disturbing the integrity of corepressor complexes (24, 33). Indeed, we found that the expression of the inactive HDAC2 isoform does not affect incorporation into the multisubunit corepressor complexes but reduces the HDAC activity and causes complex poisoning (Fig. 4). In contrast, the loss of one or two *Hdac2* alleles in the brain has no effect on the deacetylase activity of corepressor complexes, most probably due to the upregulation of the paralog HDAC1 (9) (data not shown). Interestingly, impaired HDAC2 function seems to preferentially affect the CoREST and NuRD complexes (Fig. 4) (28), while Sin3A complex activity depends more on the function of the HDAC1 enzyme (8, 10).

*Hdac2*^KI/+^ mice and *Hdac1*^Δ/+n^ *Hdac2*^Δ/Δn^ mice show a highly significant overlap of upregulated genes, including protein kinase C delta (Fig. 5). Upregulation of these genes in the presence of inactive HDAC2 was observed only in the brain and not in other tissues and was not detected in heterozygous HDAC2 knockout (*Hdac2*^+/−^) mice (Fig. 5). PKCδ plays a critical role as a mediator of apoptotic responses in various cell types, including neurons. HDAC1 and HDAC2 have been shown to repress the *Prkcd* gene in dopaminergic cell culture models, and induction of PKCδ by HDAC inhibitors sensitized dopaminergic neurons to cell death (38). Therefore, the authors of that study proposed that histone acetylation-mediated upregulation of PKC expression augments nigrostriatal dopaminergic cell death, which could contribute to the progressive neuropathogenesis of Parkinson's disease. It will be interesting to test the inducible expression of catalytically inactive HDACs in neurons of adult mice and in Parkinson's disease mouse models.

Several other members of the HDAC family exert their functions irrespective of their catalytic activity. Class IIa HDACs (HDAC4, -5, -7, and -9) have only low basal enzymatic activity and are regarded as pseudoenzymes (39). Furthermore, it has been shown that HDAC3 deacetylase-dead mutants can rescue transcriptional repression in the HDAC3-depleted mouse liver and that the deacetylase activity is dispensable for HDAC3 functions *in vivo* (16). In this study, HDAC3 was found to regulate transcription independent of its catalytic activity by interaction with NCoR and SMRT. Overexpression experiments with mutants of HDAC8 showed that phosphorylation but not enzymatic activity of the enzyme is required to protect the telomere-associated protein EST1B from ubiquitin-mediated degradation (15). In the case of the histone acetyltransferase (HAT) Gcn5, loss of HAT activity due to point mutations in the catalytic domain was not sufficient to induce the early embryonic lethality observed in Gcn5-null mice but caused cranial neural tube defects at later stages (40). This demonstrates that Gcn5 has HAT-independent functions in early mouse development and that Gcn5 acetyltransferase activity is required for neural tube closure.

Interestingly, only a few naturally occurring mutations have been identified for human HDAC1 or HDAC2 (41). For instance, frameshift mutations in the *Hdac2* gene in sporadic carcinomas with microsatellite instability and in tumors arising in individuals with hereditary nonpolyposis colorectal cancer syndrome lead to the loss of HDAC2 protein expression and enzymatic activity and render these cells more resistant to HDAC inhibitors (42). The class I deacetylase HDAC8 deacetylates cohesin, and the enzyme is implicated in Cornelia de Lange syndrome (CdLS) (43). In CdLS patients, *Hdac8* missense mutations that compromise catalytic activity have been identified, suggesting a link between the loss of HDAC8 activity and this disease (44).

In summary, our data show that HDACs have both enzymatic and nonenzymatic functions. The impact of the catalytic activity seems to be both isoform specific and cell type specific. Given that corepressor complexes contain additional enzymatic functions, such as histone demethylase and chromatin-remodeling activities (41), it will be interesting to examine in future studies the importance of enzymatic and nonenzymatic HDAC functions for these additional corepressor complex functions.

## Supplementary Material

Supplemental material
