# Supplementary material for: Essential Nonredundant Function of the Catalytic Activity of Histone Deacetylase 2 in Mouse Development
Source: Mol Cell Biol. 2016 Jan 19;36(3):462–74. doi: 10.1128/MCB.00639-15 (PMC4719423; doi:10.1128/MCB.00639-15)
Supplement: Supplemental material [file supp_36_3_462__index.html]

Essential Nonredundant Function of the Catalytic Activity of Histone Deacetylase 2 in Mouse Development — Supplemental material 

# Essential Nonredundant Function of the Catalytic Activity of Histone Deacetylase 2 in Mouse Development

## Supplemental material

- Supplemental file 1 -

  Data Set S1 (Deregulated genes in Hdac2KI/+ brains)

  XLSX, 20K
